# Psychiatric symptoms as the first manifestation of juvenile SLE complicated with Klinefelter's syndrome

**DOI:** 10.1186/1546-0096-12-S1-P316

**Published:** 2014-09-17

**Authors:** Mehrnoush Hassas Yeganeh, Reza Shiari

**Affiliations:** 1 Paediatric Rheumatology, Children's Hospital, Tehran, Iran, Islamic Republic Of

## Introduction

Systemic Lupus Erythematosus (SLE) is an autoimmune, multisystem disorder with diverse manifestations. There are limited reports on the neuropsychiatric findings as the first manifestation of systemic lupus erythematosus in children.

## Objectives

A 14-year-old Iranian boy with two years history of cognitive dysfunction, behavioural problems and recent history of epistaxis was referred to Mofid’s Children Hospital, Tehran, Iran. His work-up ended to a diagnosis of Klinefelter’s syndrome associated with juvenile systemic lupus erythematosus.

## Methods

It is a case report. It just reports a patient with unusual manifestation of systemic lupus erythematosus. The patient also had a chromosomal abnormality discovered by chromosomal study and the diagnosis of Klinefelter's syndrome was confirmed.

## Results

Patients with Klinefelter's syndrome may exhibit behavioural problems and psychological distress. These psychiatric disorders will be more prominent if be complicated with Lupus in children. In fact, psychiatric symptoms can occur as the first manifestation of juvenile SLE.

## Conclusion

In pediatric patients with psychiatric disorders, especially in those who do not respond well to the classic psychiatric treatments, a diagnosis of systemic lupus erythematosus must be strongly considered, especially if the patient has a positive family history of SLE.

## Disclosure of interest

None declared.

